# Development and psychometric properties of the Thai Graves’ ophthalmopathy quality of life (GO-QOL) questionnaire

**DOI:** 10.1186/s41687-019-0164-8

**Published:** 2019-12-31

**Authors:** Mingkwan Lumyongsatien, Benjama Keeratidamkerngsakul, Kanokrat Pornpanich, Sumalee Vangveeravong, Preamjit Saonanon, Damrong Wiwatwongwana, Pornchai Mahaisavariya, Orapan Aryasit, Krit Pongpirul

**Affiliations:** 1 Department of Ophthalmology, Mettapracharak Hospital, Sampran, District, 52 moo2 Rhikhing, Sampran, Nakhon Pathom, 73210 Thailand; 2 Department of Ophthalmology, Faculty of Medicine, Siriraj Hospital, Mahidol University, 2 Arun amarin road, Siriraj, Bangkoknoi, Bangkok, 10700 Thailand; 3 Department of Ophthalmology, Faculty of Medicine, Chulalongkorn University, 1873, Praram 4 road. Prathumwan, Bangkok, 10330 Thailand; 4 Department of Ophthalmology, Faculty of Medicine, Chiangmai University, 110, Intawaroros, Sripoom, Chiangmai, 50200 Thailand; 5 Department of Ophthalmology, Faculty of Medicine, Ramathibodi Hospital, Mahidol University, 270, Praram 6 road, Thung Phaya thai, Ratchatevi, Bangkok, 10400 Thailand; 6 Department of Ophthalmology, Prince of Songkla University, 15 Kanjanawanich road, Hatyai, Songkla 90110 Thailand; 7 Department of Preventive and Social Medicine, Faculty of Medicine, Chulalongkorn University, 1873, Praram 4 Rd. Prathumwan, Bangkok, 10330 Thailand; 8 Department of International Health, Johns Hopkins Bloomberg School of Public Health, Baltimore, MD USA; 9 Bumrungrad International Hospital, 33 Sukhumvit road. Soi3, Khlong Toei Nuea, Wattana, Bangkok, 10110 Thailand

**Keywords:** Graves’ ophthalmopathy, Thyroid, Quality of life, Questionnaire, Thai GO-QOL

## Abstract

**Purpose:**

To develop and assess the psychometric properties of the Thai version of the Graves’ Ophthalmopathy Quality of Life (GO-QOL) questionnaire.

**Background:**

Graves’ ophthalmopathy (GO) is a chronic condition that causes negative self-image and impaired visual function. These conditions impact quality of life (QOL) but are rarely documented. Graves’ Ophthalmopathy Quality of Life Questionnaire (GO-QOL) has good validity, reliability and responsiveness. In this study we developed a Thai GO-QOL questionnaire by translating the questionnaire from English to the Thai language and evaluated its reliability and validity.

**Patients and methods:**

Forward and backward translations were performed independently by four translators with extensive experience in both English and Thai. Seventy patients at the thyroid clinic responded to the Thai translated version upon their first visit and again 2–3 weeks afterwards. Validity was assessed by the content validity index (CVI) and correlation with relevant clinical parameters. Reliability was evaluated by Cronbach’s alpha, the intraclass correlation coefficient, and the Bland-Altman plot.

**Results:**

The Thai GO-QOL version showed high CVI (0.97) and a moderate negative correlation of the functional QOL score with disease severity (r = − 0.49), the clinical activity score (r = − 0.31), and exposure parameter (r = − 0.32). It showed good reliability with a high intraclass correlation coefficient (0.92) and high Cronbach’ s coefficient (0.86).

**Conclusion:**

The Thai GO-QOL has good validity and reliability. It can be used to evaluate the quality of life of Graves’ ophthalmopathy patients as a consequence of their disease in thyroid treatment programs.

## Background

Graves’ ophthalmopathy (GO) is an autoimmune orbital inflammatory disease that occurs in approximately 50% of patients with Graves’ disease [1]. The pathogenesis of the disease is associated with shared antigens and cross-reactivity of thyroid and orbital tissue. Circulating antibodies activate proteins in the extraocular muscles and orbital fat, leading to fibroblast proliferation and glycosaminoglycan production. This results in exophthalmos, orbital congestion, enlarged extraocular muscles and increased orbital fat volume, with typical effects of diplopia, and eye exposure. Infrequently, optic neuropathy develops in 5% of GO. The course of GO is commonly biphasic with an active phase characterized by orbital inflammatory signs, mostly lasting 6–24 months, followed by the inactive phase of the disease [2]. This chronic condition negatively impacts patients’ quality of life and is associated with visual impairment, psychosocial problems, and consequent disfigurement of the orbital structures. In 1997, Gerding et al. reported lower scores on the 24-Item Medical Outcomes Study Short-Form Health Survey (MOS SF-24) in GO patients compared to scores in diabetes, emphysema or heart failure patients but scores comparable to those of patients with inflammatory bowel disease [3]. The MOS SF-24 was used to evaluate the perception of general health status in GO patients in the Netherlands before a disease-specific quality of life questionnaire was proposed [3].

The first GO-specific questionnaire (GO-QOL) was developed in 1998 by Terwee et al. [4]. It contained 16 questions, 8 on visual functioning and 8 on appearance. The study showed that the GO-QOL demonstrated good validity, reliability and responsiveness [5, 6]. It also revealed good correlations with disease severity and clinical activity, even among different ethnic groups and different languages [7–10]. The GO-QOL was recommended for use by the European Group on Graves’ Orbitopathy (EUGOGO) for the assessment of clinical response parameters in clinical trials [11].

The goal of GO treatment is to improve visual function, appearance, and patients’ feelings. Thus, the self-assessment of eye condition has been recommended in the treatment program for GO patients. No health- related quality of life (HRQoL) or thyroid disease-specific questionnaire studies in GO patients have previously been conducted in Thailand. To monitor the clinical response to treatment and clinical trial outcomes, we developed a Thai version of the GO-QOL and evaluated its validity and reliability with respect to its value for Thai GO patients.

## Methods

This study was approved by the Mettapracharak Hospital Institutional Review Board and informed consent was obtained from all participants. Permission for translation was received from the proposer of the GO-QOL questionnaire (Terwee CB). Forward translation from the English language questionnaire to Thai was performed by two native Thai speakers from Chulalongkorn University with extensive experience in both the English and Thai languages. Backward translation was performed by two native speakers in English working independently. Each item in the Thai questionnaire was reviewed and troublesome items were identified by 5 oculoplastic surgeons with expertise in thyroid eye disease.

### Pretesting stage

The questionnaire was administered to 10 normal subjects, defined as healthy volunteers with no abnormal eye conditions that affected their quality of life. Discussion about the meaning and clarity of each item was performed.

### Testing stage

We enrolled 70 consecutive participants with Graves’ ophthalmopathy (age > 18) at the oculoplastic clinic in Mettapracharak Hospital. Only patients who understood Thai were enrolled. GO patients whose quality of life could be affected by other eye diseases were excluded. Seventy participants completed the Thai-translated questionnaires twice. The first time was at the clinic, and the second was 2–3 weeks later. The short period was intended to prevent recall bias and changes in the stability of clinical signs. All patients were interviewed to determine whether any items were difficult or confusing to answer or irrelevant to the disease.

The Thai version of the GO-QOL contained 16 items with 8 questions pertaining to the consequences of diplopia and decreased vision on visual functioning and 8 questions on the psychosocial consequences of a changed appearance [6]. Each item was answered on a 3-point Likert scale (1, “seriously limited”; 2, “a little limited”; 3, “not limited at all”). The answer scores for items 1–8 and for items 9–16 were tallied to provide 2 raw scores. Each possible subscale score ranged from 8 to 24; the first one was a functioning subscale, and the second one was an appearance subscale. The two raw subscale scores were transformed to 2 total scores by the following formula: total score = (raw score - Y) / (2 x Y) × 100, where Y is the number of completed question items. Each total score ranged from 0 to 100, with lower scores representing poorer health status. If participants did not complete any item for any reason, such as “never learned to ride a bike” or “have no driver’s license”, the missing item could be excluded, leaving the remaining completed items for calculation [6, 12].

Patients’ demographic data were collected. Current GO severity was graded by the EUGOGO classification system, which divides patients into mild, moderate to severe and very severe groups. Soft tissue inflammation and activity was graded by clinical activity scores based on inflammatory signs of the orbit (pain, redness and swelling).

Validation focused on content and construct validity. Content validity involved the systematic examination of the item content to determine whether it was applicable to, relevant to and reflective of Thai GO patients and was determined using a content validity index (CVI). Each item was rated by five experts on a 4-point ordinal scale (grade 1, not relevant; grade 2, somewhat relevant; grade 3, quite relevant; grade 4, highly relevant). The CVI was assessed both at the item level (I-CVI) and the scale level (S-CVI). The item level (I-CVI) was calculated as the following formula: I-CVI = N_R_ / N, where N_R_ is the number of experts giving a score of grade 3 or grade 4 and N is the total number of experts [13]. I-CVI should be at least 0.78 for sufficient agreement [13]. The scale level CVI (S-CVI) was computed with 2 different indices: 1) universal agreement (S-CVI/UA) was calculated by the formula S-CVI/UA = A / B, where A is the number of items that were judged relevant to grade3 or grade 4 by all experts (I-CVI = 1.0), and B is the number of total items, and 2) average agreement (S-CVI/Ave) was computed by summing all I-CVI and dividing by the number of total items [14]. An acceptable S-CVI should be at least 0.8 [14, 15].

Construct validity refers to how well a test measures its intended construct [16]. This was assessed by evaluating specific hypotheses through the correlation between QOL scores and other clinical parameters. The functioning subscale had moderate negative relationships with disease severity and clinical activity score (CAS) values. The appearance subscale was weakly to moderately correlated with four measures: 1) age, with younger patients reporting more problems with appearance than older patients; 2) sex, with female patients reporting more problems with appearance than men; 3) GO disease severity; and 4) eye exposure parameters, with scores worsening as eye exposure increased. The expected magnitude of the differences in GO-QOL scores between various severity groups was at least 10 points as a minimal clinically important difference [5]. The criteria for good construct validity are defined as at least 75% of the results are coherent with the hypotheses [17]. A one-way analysis of variance (ANOVA) was used to identify differences in the mean QOL scores between the various severity groups. Spearman’s rank correlation coefficient was performed to assess the correlation between disease severity, CAS and eye exposure parameters with QOL scores. The internal consistency of the questionnaires was assessed by calculating Cronbach’s alpha. Factor analysis was performed to categorize 16 items of the Thai GO-QOL into group domains; the cutoff point of factor loading was 0.4 [18]. Test-retest reliability was evaluated according to the intraclass correlation coefficient (ICC) and Bland-Altman plot [19]. A two-way mixed model for absolute agreement was used to calculate the ICC [20]. All data were analyzed using SPSS version 21.0 software (SPSS Inc., Chicago, IL, USA).

## Results

### Results of pretesting

Ten normal participants reported that the Thai GO-QOL questionnaire was not difficult to complete or confusing.

### Results of testing

All 70 participants were recruited and completed every item of the Thai version of the GO-QOL. They reported that the questionnaire was not difficult, confusing or irrelevant to the disease. Table 1 shows the demographic data and clinical characteristics of the GO patients. Most of the participants were female. The mean age was 49.4 ± 12.1 years. Diplopia and dry eye were the most common current symptoms (36.6%). Approximately two-thirds of the patients had moderate to severe severity (67.1%). Six patients (8.6%) had dysthyroid optic neuropathy. The average CAS was 1.0, and 6 patients (8.6%) had active disease.

**Table 1:** Participants’ demographic data (*n* = 70)

Variable	Value
Age (years)	49.4 ± 12.1 (24–81)
Gender (F:M)	40:30
Smoking	8 (11.4%)
Associated thyroid disease	
Graves’ disease	66 (94.3%)
Hashimoto thyroiditis	1 (1.4%)
Hypothyroid	1 (1.4%)
None	2 (2.8%)
Current thyroid status	
Hyperthyroid	8 (11.4%)
Hypothyroid	6 (8.6%)
Subclinical hyperthyroid	4 (5.7%)
Subclinical hypothyroid	3 (4.3%)
Euthyroid	49 (70%)
Treatment thyroid disease	
< 1 treatment	59 (84.3%)
Antithyroid drug	33 (47.1%)
Radioiodine	2 (2.9%)
Thyroidectomy	1 (1.4%)
Thyroxine	9 (12.9%)
None	14 (20.0%)
> 1 treatments	11 (15.7%)
Antithyroid drug, radioiodine	1 (1.4%)
Thyroidectomy, antithyroid drug	2 (2.9%)
Thyroidectomy, thyroxine	2 (2.9%)
Radioiodine, thyroxine	6 (8.5%)
Other autoimmun	2 (2.8%)
Steroid treatment for GO	
IVMP	16 (37.2%)
Oral steroid	7 (16.3%)
Orbitotomy	20 (46.5%)
Current symptoms	
Orbital pain	6 (5.9%)
Pain on eye movement	2 (1.9%)
Dry eye	37 (36.6%)
Photophobia	3 (2.9%)
Watery	7 (6.9%)
Diplopia	37 (36.6%)
Blurred vision	9 (8.9%)
Active GO (CAS > 3)	6 (8.6%)
CAS	1 ± 1.2 (0–5)
GO severity by EUGOGO classification	
Mild	17 (24.3%)
Moderate to severe	47 (67.1%)
Very severe (DON)	6 (8.6%)
Visual acuity (best corrected VA)	
Right eye	0.6 ± 0.2 (0–1)
Left eye	0.6 ± 0.2 (0.03–1)
Exophthalmos (mm.)	
Right eye	18.0 ± 3.3 (10–25)
Left eye	18.2 ± 3.2 (9–25)
Lid retraction (*N* = 44)	
Upper lid (MRD1 minus 5)	2.7 ± 0.64
Lower lid (from limbus)	1.7 ± 0.93
Occupation	
Agriculture	6 (8.6%)
Employer	28 (40%)
Employee	15 (21.4%)
Homemaker	14 (20%)
Retirement	4 (5.7%)
Not working related to eye condition	2 (2.8%)

**Fig. 1:**
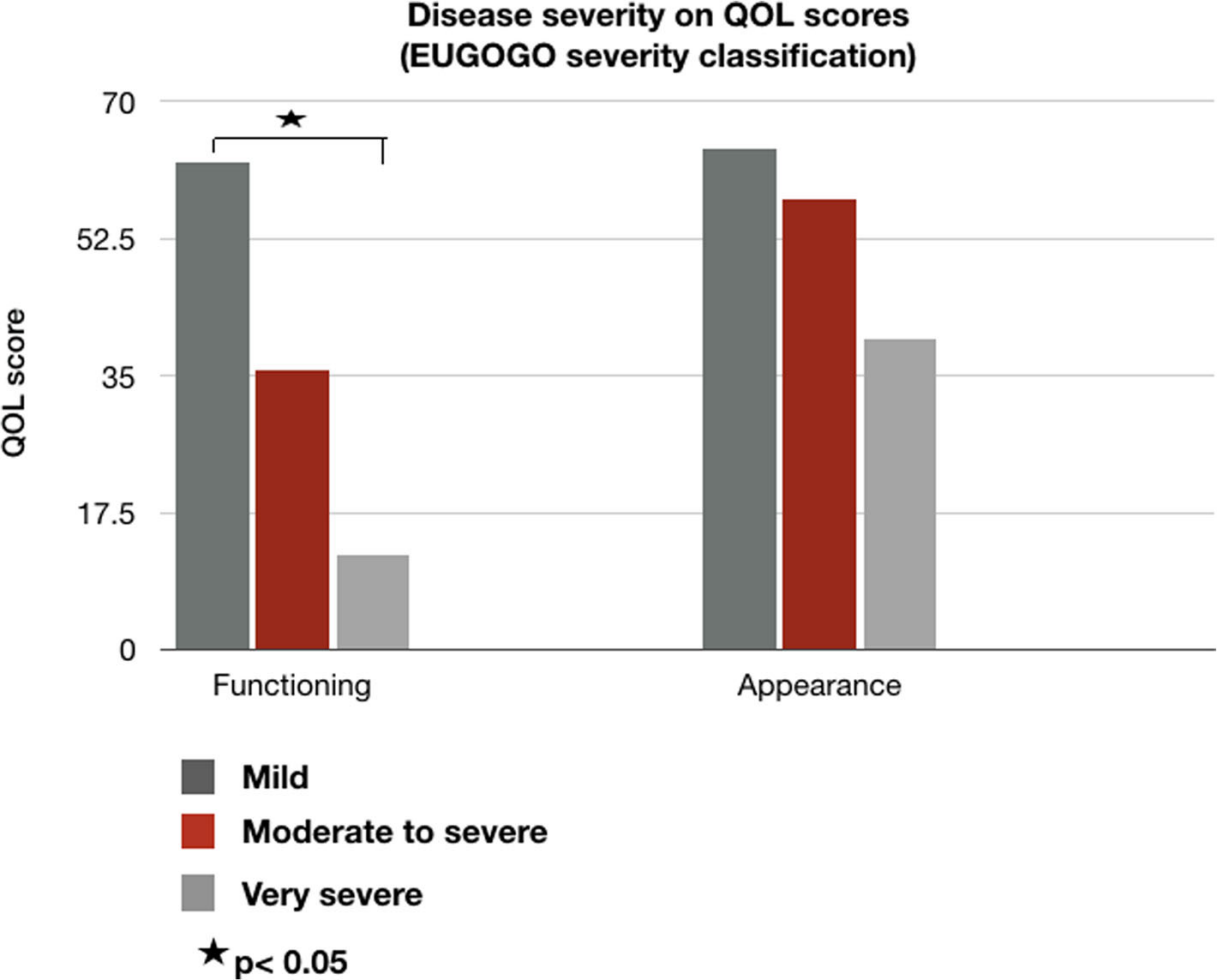
Disease severity on means of adjusted QOL scores

The frequencies of responses on the Thai GO-QOL are presented in Table 2. The percentage of completed responses for each item was 100%. Seriously limited activities were described by 48.6% of patients for reading, by 42.9% for driving, by 44.3% for interference with daily life, and by 40% for walking outdoors and watching TV. Most patients (95.7%) felt that their appearance had changed (a little or very much), 75.7% felt the influence of the disease on their self-confidence (a little or very much), and 71.4% felt that they were stared at on the street (a little or very much). The average score for visual functioning was 40.17 ± 28.65 (mean ± SD). The average appearance score was 57.50 ± 26.76.

**Table 2:** Frequencies of responses on the visual functioning and appearance subscale of the Thai GO-QOL (*N* = 70)

Item	*Seriously limited (%)*	*A little limited (%)*	*Not limited (%)*	*Missing response (%)*
Functioning				
1. Bicycling	31.4	30.0	22.9	15.7(a)
2. Driving	42.9	24.3	17.1	15.7(b)
3. Moving around the house	25.7	45.7	28.6	0.0
4. Walking outside the house	40.0	34.3	25.7	0.0
5. Reading	48.6	37.1	14.3	0.0
6. Watching TV	40.0	44.3	15.7	0.0
7. Enjoying hobby or pastime	32.9	52.9	14.3	0.0
8. Prevented from doing what you want to do	44.3	40.0	15.7	0.0
*Appearance*	*Very much (%)*	*A little (%)*	*No (%)*	*Missing response (%)*
9. Changed physical appearance	45.7	50.0	4.3	0.0
10. Stared at on the streets	37.1	34.3	28.6	0.0
11. People have a negative reaction	10.0	30.0	60.0	0.0
12. Influence on self-confidence	47.1	28.6	24.3	0.0
13. Social isolation	7.1	20.0	72.9	0.0
14. Effect on making friends	8.6	22.9	68.6	0.0
15. Reluctance to be photographed	34.3	32.9	32.9	0.0
16. Hide or conceal physical changes	25.7	30.0	44.3	0.0
(a) “never learned to ride a bike” 15.7% (b) “no driver’s license” 15.7%

**Table 3:** Rating on 16 items of QOL by five experts: Content validity index

QO-QoL Questionnaire	Relevant (grade 3 or 4)	Not relevant (grade 1 or 2)	Content validity index (item CVI)
Q1	4	1	0.8
Q2	4	1	0.8
Q3	5	0	1.0
Q4	5	0	1.0
Q5	5	0	1.0
Q6	5	0	1.0
Q7	5	0	1.0
Q8	5	0	1.0
Functioning	38	2	0.95
Q9	5	0	1.0
Q10	5	0	1.0
Q11	5	0	1.0
Q12	5	0	1.0
Q13	5	0	1.0
Q14	5	0	1.0
Q15	5	0	1.0
Q16	5	0	1.0
Appearance	40	0	1.0
Total	78	2	0.97
		S-CVI/Ave	0.97
		S-CVI/UA	0.87

S-CVI/Ave, Scale-content validity index, averaging calculation method

S-CVI/UA, Scale-content validity index, universal agreement calculation method

### Validity

There were high content validity indices for each item question (I-CVI > 0.8) and the mean of all items (S-CVI/Ave =0.97) (Table 3). The visual functioning scores were moderately negatively correlated with disease severity (r = − 0.49), CAS (r = − 0.31) and lid retraction (r = − 0.32). The appearance scores were weakly negatively correlated with disease severity (r = − 0.20) and dry eye severity (r = − 0.24). Age was weakly correlated with QOL scores, while female sex was not correlated with the scores (Table 4). The mean visual functioning in each severity group was statistically significantly different (*p* < 0.001, *p* = 0.01) (Table 5 and Fig. 1). In accordance with the hypotheses, the construct validity of the visual functioning subscale was 100% (3 of 3 criteria), and that of the appearance subscale was 80% (4 of 5 criteria) (Tables 4 and 5).

**Table 4:** Correlation between QOL score, clinical activity score, disease severity, age, sex and exposure (*n* = 70)

	QOL score	
	Functioning	Appearance
Age	−0.13	0.16
Sex (female)	−0.04	0.09
CAS	−0.31	−0.05
Severity (EUGOGO classification)	− 0.49	− 0.20
Exposure/Appearance		
Proptosis	−0.02	− 0.14
Lid retraction	−0.32	− 0.12
Dry eye	−0.07	−0.24

Data expressed as correlation coefficient and calculated by Spearman rank correlation coefficient, point biserial correlation for sex

**Table 5:** Disease severity on means of adjusted QOL scores and differences between groups

		*Clinical severity*		
*QOL score*	*Mild severity (N = 17) (mean ± SD)*	*Moderate to severe severity (N = 47) (mean ± SD)*	*Very severe severity (N = 6) (mean ± SD)*	*P value*
Functioning	62.25 ± 23.33	35.76 ± 26.72	12.15 ± 15.50	< 0.001
Appearance	63.97 ± 19.58	57.45 ± 29.30	39.58 ± 15.13	0.159

### Reliability

The results of the factor analysis are presented in Table 6. Factor analysis with varimax rotation was used to categorize the 16 items of the questionnaire into four group factors, explaining 72.93% of the total variance. Items that loaded high on the first factor were related to problems with near to intermediate vision. Items that loaded high on the second factor were associated with psychosocial problems. Items that loaded high on the third factor were correlated with changed appearance, and items that loaded high on the fourth factor were related to trouble with distant vision. A two-factor structure confirmed the subdivision of the questionnaire into 2 subscales for visual functioning (near to distance vision) and the psychosocial effects of changed appearance.

**Table 6:** Factor analysis with varimax rotation of the 16 items of the Thai GO-QOL

		*4 Factors*	*result*		*2 Factors*	*result*
*Thai GO-QOL*	*Factor 1*	*Factor 2*	*Factor 3*	*Factor 4*	*Factor 1*	*Factor 2*
Question 1	0.17	0.11	0.04	**0.89**	0.10	**0.54**
Question 2	0.34	−0.01	0.03	**0.82**	0.02	**0.65**
Question 3	**0.70**	0.34	0.08	0.31	0.29	**0.79**
Question 4	**0.74**	0.15	0.28	0.24	0.32	**0.75**
Question 5	**0.78**	0.11	0.08	−0.03	0.15	**0.67**
Question 6	**0.85**	0.10	0.03	0.03	0.10	**0.77**
Question 7	**0.76**	0.05	−0.09	0.20	−0.02	**0.78**
Question 8	**0.62**	0.18	0.08	0.27	0.19	**0.68**
Question 9	−0.14	0.09	**0.85**	−0.01	**0.71**	−0.21
Question 10	0.01	0.39	**0.75**	0.24	**0.83**	0.07
Question 11	0.15	**0.85**	0.19	0.11	**0.70**	0.26
Question 12	0.30	0.12	**0.77**	−0.12	**0.60**	0.13
Question 13	0.18	**0.86**	0.16	0.02	**0.68**	0.25
Question 14	0.19	**0.87**	0.20	0.07	**0.72**	0.28
Question 15	0.12	0.34	**0.69**	0.29	**0.75**	0.20
Question 16	0.22	0.48	**0.56**	−0.15	**0.74**	0.12
**Eigenvalues**	6.09	2.78	1.42	1.35	6.09	2.78
**% of Variance**	38.12	17.43	8.91	8.47	38.12	17.43
**Cumulative%**	38.12	55.55	66.46	72.93	38.12	55.55

Eigenvalues = the total variance explained by each factor

% of Variance = percentage of the total variance explained by each factor

Boldface numbers = high factor loading

Cronbach’s alphas were 0.86 for visual functioning and 0.87 for appearance. The intraclass correlation coefficients were 0.92 (95% CI, 0.88–0.95) for visual functioning and 0.90 (95% CI, 0.85–0.94) for appearance scores (Table 7).

**Table 7:** Cronbach’s alpha and intraclass correlations for test-retest data

Scale	Cronbach’s alpha	Intraclass correlation (95% CI)
Functioning	0.86	0.92 (0.88–0.95)
Appearance	0.87	0.90 (0.85–0.94)

**Fig. 2:**
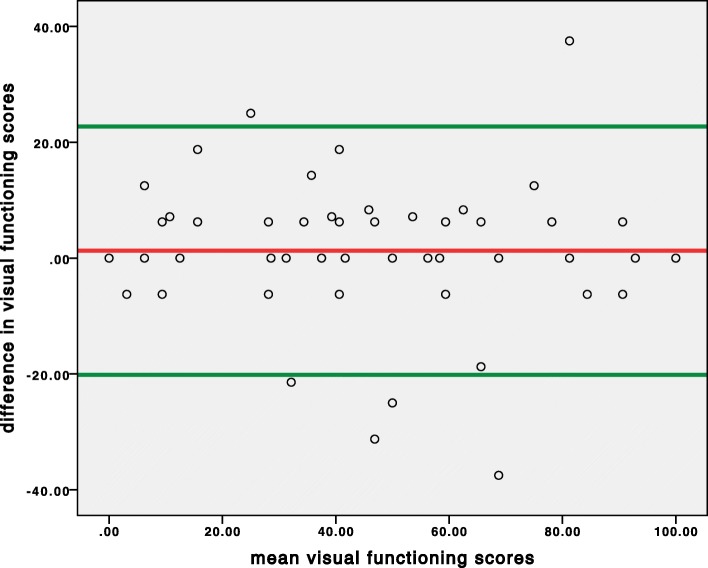
Bland-Altman plot analysis for agreement between the first and the second measurement of the functioning subscale scores

**Fig. 3:**
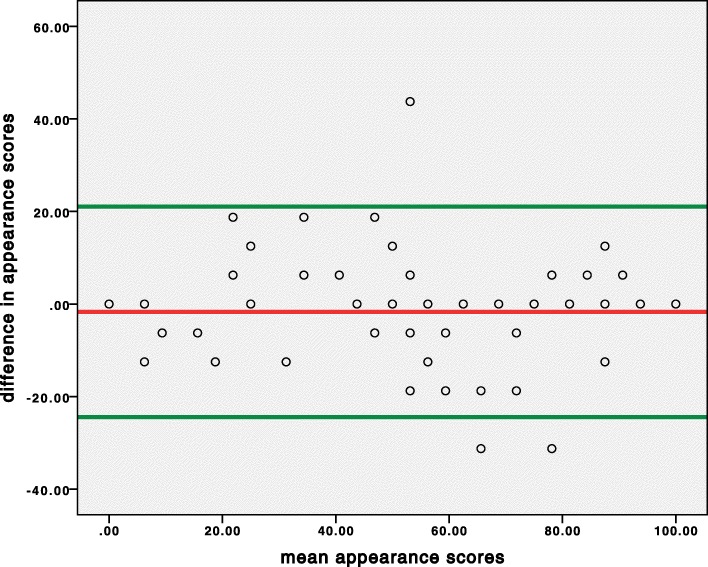
Bland-Altman plot analysis for agreement between the first and the second measurement of the appearance subscale scores

Figures 2 and 3 display a scatter diagram of the differences between the first and second measurements plotted against their means, with a presentation of the limits of agreement (mean difference ± 1.96 SD) at 1.27 ± 21.42 for the functioning subscale and − 1.69 ± 22.73 for the appearance subscale.

## Discussion

The results of this study showed good reliability and validity of the Thai version of the GO-QOL. High Cronbach’s alpha and intraclass correlation coefficients suggested good reliability and internal consistency of the questionnaire. The Bland-Altman plot also confirmed the repeatability of the questionnaire in both subscales; this result corresponded to the original study by Terwee [7]. The high content validity index of each item and the average of all items supported good content validity, whereas construct validity was supported by the correlation of QOL scores with disease severity, clinical activity scores, and exposure parameters. There were significant differences in the means of visual functioning scores among the varying severity groups. Interestingly, the appearance subscale score was weakly correlated with disease severity. This result was similar to the original study by Terwee et al. [4]. The mean appearance scores for the different severity groups were not significantly different, suggesting that GO severity might have less influence than individual perception on changing appearance.

Gerding et al. reported that the HRQoL scores in GO patients did not correlate with the duration, severity or activity of the disease. They concluded that usual clinical assessment seems to be unrelated to the negative impact on quality of life [3].

Regarding well-being among Graves’ disease patients with or without ophthalmopathy assessed by HRQoL, GO-QOL and Mini-Mental State Examination, Riguetto CM et al. found that the presence of ophthalmopathy was a factor related to poor quality of life [21].

From the perspective of patients, clinical measurements, such as extraocular muscle movement or the degree of proptosis, are of limited interest. Instead, patients usually consider to impaired physical and psychosocial issues in daily life [6, 22]. The difference between objective clinical measurements and patients’ experiences cannot be explained only by the severity of signs and symptoms but by individuals’ characteristics and the environment, such as expectations, motives, past experiences, stress coping, doctor-patient relationships and social support [22]. Health-related quality of life is the most important indicator of successful treatment when the primary aim is to improve quality of life rather than to prolong life [23].

Previous GO-specific quality of life studies have shown only a moderate correlation between QOL and disease severity and a low correlation in appearance subscales [4, 9, 16, 24, 25]. This evidence accentuates the disparity between objective clinical assessment and subjective quality of life; hence, assessing both objective and subjective measurements is the best approach for GO treatment programs [16].

There are some limitations to this study. First the translators had no background in medicine or understanding of GO. With regard to some ambiguous terms referring to GO in the Thai language, the translators were informed of concepts included in the questionnaire during the first stage of translation. The expert committee worked closely with all the translators in the process.

Content validity refers to the relevance, comprehensiveness and comprehensibility of a questionnaire [26]. It can be evaluated by asking experts and patients [26]. In this study, relevance was evaluated among experts and GO patients; comprehensibility was evaluated among ‘normal subjects’ and patients. However, as another limitation of the study, comprehensiveness was not adequately addressed.

## Conclusion

The Thai GO-QOL questionnaire indicates good reliability and validity similar to its prototype. Its scores correlated with clinical activity, disease severity and eye exposure parameters. The Thai version of the GO-QOL can be implemented into thyroid disease treatment programs to evaluate dynamic clinical outcome measurements of Graves’ ophthalmopathy.

## Abbreviations

CAS: clinical activity score
CVI: content validity index
EUGOGO: European Group on Graves’ Orbitopathy
GO: Graves’ ophthalmopathy
MOS SF-24: 24-item Medical Outcome Study Short-Form Health Survey
QOL: quality of life
